# RIGA-FEDE-LIKE DISEASE IN A 70 YEAR OLD WOMAN

**DOI:** 10.4103/0019-5154.60361

**Published:** 2010

**Authors:** Uwe Wollina

**Affiliations:** *From the Department of Dermatology and Allergology, Hospital Dresden-Friedrichstadt, Academic Teaching Hospital of the Technical University of Dresden, Dresden, Germany.*

**Keywords:** *Riga-Fede-disease*, *traumatic tongue ulcer*, *prosthesis*

## Abstract

Riga-Fede disease (RFD) describes a benign, ulcerative lesion resulting from the repetitive trauma of contact of the oral mucosal surface of the tongue with the teeth. Although the name applies primarily to small children, similar clinical and histopathological findings can also be found in adults. We describe here a 70 year-old woman showing a painful tongue ulcer with elevated borders and whitish discoloration for the past four years. Repeated histological investigations revealed a benign leukoplakia without dysplasia. Replacement of an ill-fitting prosthesis led to complete remission within two weeks. RDF-like disease is thus a problem in elderly patients for whom topical treatment is insufficient to induce healing.

## Introduction

Riga-Fede disease (RFD) describes a benign, ulcerative “granulomatous” lesion resulting from the repetitive trauma of contact of the oral mucosal surface of the tongue with the teeth. This entity was first described in 1881 by the Italian physician, Antonio Riga, and as additional cases were subsequently published by F. Fede in 1890, it has been known as Riga-Fede disease.[1,2] Although the name applies primarily to children, similar clinical and histopathological findings can also be found in adults.[3,4] Although the lesions are microscopically identical, the causes of trauma differ in the adult population as they may be related to the presence of broken teeth or ill-fitting prosthetic material in the oral cavity. Early recognition of this entity is important because it may be the presenting sign of an underlying neurological disorder.[1]

This condition has rarely been described in elderly people, which is surprising as denture-related lesions account for 36.4% of the cases with the length of denture use and diabetes mellitus being significant risk factors for denture stomatitis and denture hyperplasia.[5]

A broad variety of terms have been used to describe RFD, such as eosinophilic ulcer of the oral mucosa, sublingual fibrogranuloma, sublingual growth in infants, lingual traumatic ulceration, traumatic atrophic glossitis, traumatic granuloma of the tongue, and traumatic ulcerative granuloma with stromal eosinophilia.[1–4,6,7]

RFD-associated ulceration may remain for years and can result in long-lasting tongue deformity.[1,2]

## Case Report

A 70 year-old woman presented with a painful ulcer with elevated borders and whitish discoloration. The lesion had been present for the past four years and had been biopsied twice. Histological investigations revealed a benign leukoplakia of the tongue without dysplasia. Treatment was done with topical corticosteroids but failed to induce complete remission. The patient had no other systemic medications.

**Figure 1:**
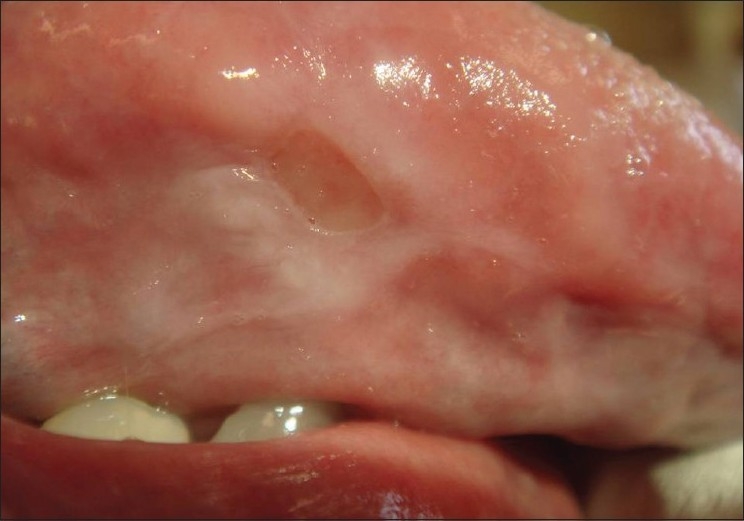
Painful ulcer of the tongue, Riga-Fede disease-like

**Figure 2:**
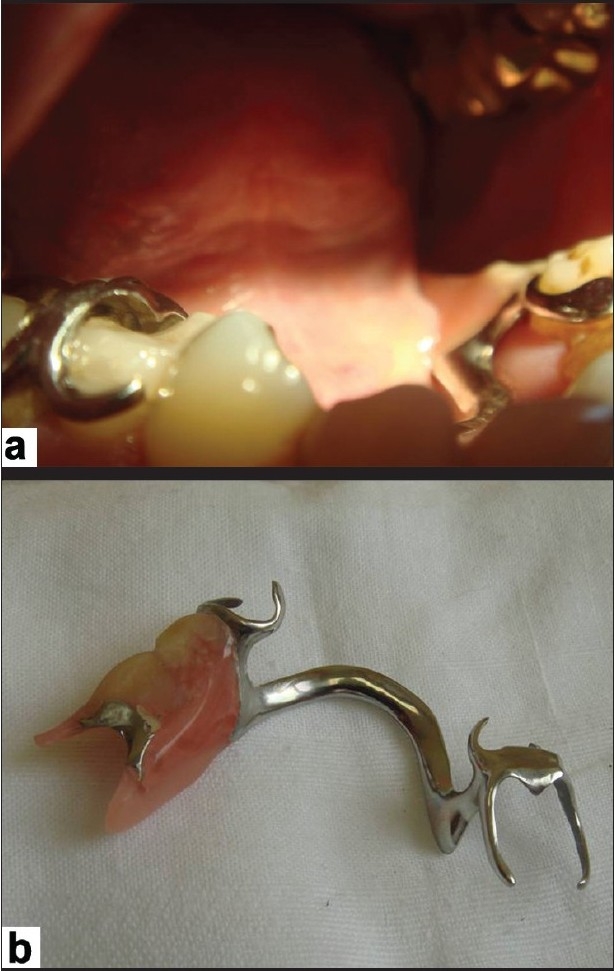
Ill-fitting prosthesis. (a) *In situ*. (b) Detail

**Figure 3:**
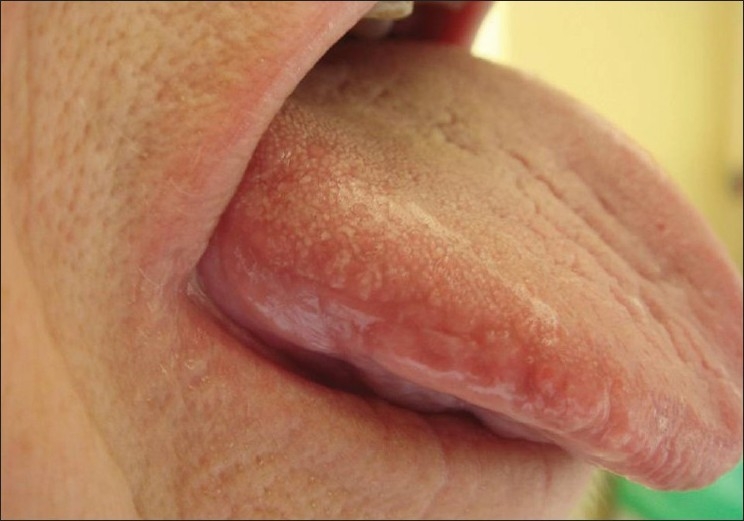
Complete healing within two weeks without the prosthesis

The patient was subsequently referred to our outpatient department for further advice. Examination of the oral cavity revealed an ulceration of the left undersurface of the tongue [Figure 1] and showed close contact with an ill-fitting prosthesis of the mandibular molars [Figure 2]. There was no clinical sign of any neuropathy and the patient was advised not to use the prosthetics for two weeks and to come back to the department at the end of this period. Complete healing was observed at that time [Figure 3] and a relapse of the ulcer was noted after another week of use of the prosthesis.

A diagnosis of a chronic traumatic RFD-like tongue ulcer was confirmed and the patient was referred to her dentists to improve the fitting of her prosthesis.

## Discussion

Chronic ulcerations of the tongue are not uncommon in old age. The differential diagnoses include various benign and malignant conditions: Squamous cell carcinomatous ulcer, syphilis chancre, tuberculosis, malakoplakia, cytomegalovirus infection, fungal disease, major aphthous ulcer, Behcet's disease, mucous membrane pemphigoid, pyoderma gangraenosum, orofacial granulomatosis, graft-versus-host disease, ulcerating lichen planus mucosae, amyloidosis, autoaggression, midline granuloma, necrotizing sialometaplasia, lymphoma and leukemia.[1,2,6–19] Drugs, particularly chemotherapeutics and radiotherapy can cause chronic ulcerations of the tongue as well. Less well known is the development of chronic tongue ulcers in bisphosphonate-associated osteonecrosis of the jaws.[20] Hence, diagnosis must be based on histopathological findings and course of the disease.

Histological investigations show a chronic mucous ulceration surrounded by a dense infiltrate of lymphocytes, monocytes, and numerous eosinophils in RFD and RFD-like conditions, without any other findings related to infection or neoplasia. Although clinically described as a granulomatous process, no granuloma has been seen microscopically.[1,2]

Treatment is aimed at minimizing the repetitive trauma and consists of behavior modification and dental extraction, filling of the teeth, or acrylic appliances placed over the broken teeth.[1,2,20] If ill-fitting prosthetic material is the cause of trauma, it has to be exchanged as in the presented case. Complete remission is achievable only when the provoking factors can be avoided.
